# Association of serum transforming growth factor β_1_ with left ventricular hypertrophy in children with primary hypertension

**DOI:** 10.1007/s00431-023-05219-2

**Published:** 2023-09-27

**Authors:** Yang Liu, Yao Lin, Xiaolan Huang, Yaqi Li, Yanyan Liu, Lin Shi

**Affiliations:** 1 Department of Cardiology, Children’s Hospital, Capital Institute of Pediatrics, Beijing, China; 2 Central Laboratory, Capital Institute of Pediatrics, Beijing, China

**Keywords:** Hypertension, Children, Serum transforming growth factor β_1_, Body mass index, Left ventricular hypertrophy

## Abstract

The current study was designed to assess the association of serum transforming growth factor β_1_ (TGF-β_1_) with left ventricular hypertrophy (LVH) in children with primary hypertension. The present single-center prospective trial examined 182 patients diagnosed with primary hypertension in Children’s Hospital, Capital Institute of Pediatrics, between January 2021 and September 2022. Clinical data were analyzed, and ambulatory blood pressure was assessed for 24 h. LVH, the commonest subclinical cardiac feature of hypertension, was assessed by echocardiography. According to left ventricular geometry, cases were assigned to the LVH (*n* = 44) and normal geometry (*n* = 138) groups. Serum TGF-β_1_ amounts were quantitated by enzyme-linked immunosorbent assay (ELISA). Receiver operating characteristic (ROC) curves were established to analyze various variables for their predictive values in LVH. Among 182 children with primary hypertension, the concentrations of serum TGF-β_1_ were higher in stage 2 hypertension than in stage 1 (47.3 (38.8, 52.5) *vs.* 46.0 (38.6, 48.2) ng/L, *Z* =  − 2.376; *P* = 0.018). Additionally, serum TGF-β_1_ content showed a positive correlation with BP levels (*P* < 0.05). TGF-β_1_ amounts were significantly elevated in the LVH group compared with the normal geometry group (51.7 (46.1, 54.9) vs. 46.1 (38.7, 48.1) ng/L, *Z* =  − 4.324; *P* = 0.0000). Serum TGF-β_1_ content was positively associated with LVH (*r* = 0.321, *P* = 0.0000). Multivariable logistic regression analysis showed BMI (OR = 1.188, 95% CI 1.082–1.305; *P* = 0.0000) and elevated serum TGF-β_1_ content (OR = 1.063, 95% CI 1.016–1.113; *P* = 0.009) independently predicted LVH. A multivariable logistic regression model considering BMI and TGF-β_1_ content in LVH prediction was 0.771, with sensitivity and specificity of 72.7% and 70.3%, respectively.

*Conclusion*: These data revealed an association of serum TGF-β_1_ with BP in children with primary hypertension. Serum TGF-β_1_ concentration was positively correlated with hypertensive cardiac damage. Serum TGF-β_1_ might constitute a valuable molecular marker for the prediction of LVH in children with primary hypertension. The combination of BMI and TGF-β_1_ has a certain diagnostic and predictive value for LVH in children with primary hypertension, which may provide a new reference index for early clinical identification of hypertensive cardiac damage.

**Table:** 

**What is Known:** *• Experimental and clinical data indicated TGF-β1 is involved in BP elevation.* *• TGF-β1 is positively correlated with LVMI and hypertrophy in adults.*	
**What is New:** *• Our current study reveals an association of serum TGF-β1 with BP in children with primary hypertension.* *• Elevated serum TGF-β1 level is positively associated with LVH in children with primary hypertension.* *• The combination of BMI and TGF-β1 has a certain diagnostic and predictive value for LVH in children with primary hypertension.*

**What is Known:**

*• Experimental and clinical data indicated TGF-β1 is involved in BP elevation.*

*• TGF-β1 is positively correlated with LVMI and hypertrophy in adults.*

**What is New:**

*• Our current study reveals an association of serum TGF-β1 with BP in children with primary hypertension.*

*• Elevated serum TGF-β1 level is positively associated with LVH in children with primary hypertension.*

*• The combination of BMI and TGF-β1 has a certain diagnostic and predictive value for LVH in children with primary hypertension.*

## Introduction

Transforming growth factor β_1_ (TGF-β_1_), a cytokine with multiple functions, belongs to the TGF-β superfamily expressed by vascular wall cells [1, 2]. TGF-β_1_ regulates cell growth, differentiation, and growth of extracellular matrix, which is produced by myocardial cells, vascular smooth muscle cells, endothelial cells, macrophages, etc. TGF-β_1_ is very important in hypertensive vascular remodeling, by affecting hemodynamics in the body, stimulating cytoendothelin production or renin-angiotensin-aldosterone system (RAAS) activation [3, 4]. Therefore, current studies on adults believe that TGF-β_1_ is tightly associated with the pathogenesis of hypertensive target organ damage (TOD) [5].

Recently, an increasing incidence of pediatric primary hypertension has been reported. Although cardiovascular events rarely occur, subclinical TOD is common [6–8], and the associated damages can persist into adulthood, putting these patients at high risk of severe cardiovascular diseases [9]. Lili Y et al. [10] showed that elevated blood pressure in children and adolescents was significantly correlated with high pulse wave velocity (PWV), high carotid artery intima-media thickness (cIMT), and left ventricular hypertrophy (LVH), which was also strongly associated with cardiovascular events and all-cause mortality in adulthood. This study further supported the adverse effects of elevated blood pressure in children and adolescents on cardiovascular health in adulthood. In recent years, studies have shown that 30% of children with hypertension had LVH in the early stages [11]. The evaluation of left ventricular mass (LVM) and ventricular configuration is currently considered the basis for assessing early cardiac damage in children with hypertension [12, 13]. As an intermediate stage in the progression of hypertensive cardiac damage from childhood to adulthood, early cardiac assessment has important values [10]. Therefore, the early recognition of LVH is of great significance for the early prevention and treatment of hypertension and cardiovascular diseases in children. TGF-β_1_ amounts were shown to be positively correlated with blood pressure (BP) in adult patients [14]. However, previous studies have not examined serum TGF-β_1_ amounts in pediatric primary hypertension cases. The current study aimed to assess the association of serum TGF-β_1_ with -LVH in children with primary hypertension.

## Patients and methods

### Subjects

The present single-center prospective trial enrolled 182 pediatric patients who were first diagnosed with primary hypertension hospitalized in the Children’s Hospital, Capital Institute of Pediatrics (CIP; Beijing, China), between January 2021 and September 2022, including 140 males (76.9%) and 42 females (23.1%). This trial had approval from the Ethics Committee of the CIP (No: SHERLL2021021). The parents/guardians of all patients provided signed informed consent prior to enrolment.

The diagnosis was made following the indications proposed in the Chinese guidelines for the diagnosis and management of hypertension in children and adolescents [15], and all blood pressure measurements were performed using the auscultation method as recommended [16]. Hypertension diagnosis was reflected by average systolic BP (SBP) and/or diastolic BP (DBP) beyond the 95th percentiles of the auscultation measurement, after adjustments for gender, age, and height. Stages 1 and 2 hypertension were diagnosed with BP < 99th and ≥ 99th percentiles, respectively, plus 5 mmHg.

Exclusion criteria were age > 18 years; secondary hypertension associated with kidney, vascular, endocrine, or central nervous system (CNS) disease or drug treatment; and primary hypertension previously treated with antihypertensives.

### Demographic and laboratory data

Demographic data include age, gender, height, weight, and body mass index (BMI) [BMI= weight (kg)/height^2^ (m^2^)]. The diagnostic criteria for obesity is BMI ≥ 95th percentiles, after adjustments for gender and age [17]. Laboratory data include fasting blood glucose, blood lipids, plasma renin activity, angiotensin II, aldosterone, and other biochemical indicators. Biochemical indexes were quantitated in plasma or serum by routine assays.

### Serum TGF-β_1_ measurement

Serum human TGF-β_1_ was detected by enzyme-linked immunosorbent assay (ELISA) with a specific kit (R&D Systems, USA) as directed by the manufacturer.

### Blood pressure measurements

The pediatric cases were submitted to 24-h ambulatory blood pressure monitoring (ABPM) with a DMS-ABP device (DM Software, China). After training, the patients measured their BP themselves. Although daily activities were allowed, the patients were instructed to perform measurements in the resting state at 30-min and 60-min intervals in the daytime and during sleep, respectively. Nighttime was defined by recording BP in the sleep and wake periods and adjusting the values for every individual. Accordingly, 24-h SBP, 24-h DBP, daytime and nighttime SBP, and daytime and nighttime DBP were assessed. Method reliability was reflected by > 70% valid measurements [18].

### Left ventricular hypertrophy and classification

LVH was assessed by echocardiography. A Philips iE33 Ultrasound System (Philips Healthcare, USA) was utilized to measure left ventricular internal dimension (LVIDd), interventricular septal thickness (IVST), and left ventricular posterior wall thickness (LVPWT) at the end of diastole. LVM was derived as 1.04 × 0.8 × ((LVIDd + IVST + LVPWT)^3^ – LVIDd^3^) + 0.6 [19]; LVM index (LVMI) was determined as LVM/height^2.7^. Relative left ventricular wall thickness (RWT) was derived as (IVST + LVPWT)/LVIDd. We adopted the criteria proposed by Hietalampi et al. for the diagnosis of abnormal cardiac geometry [20], namely, LVMI ≥ 37.08 g/m^2.7^ and 34.02 g/m^2.7^ in boys and girls, respectively; (b) RWT > 0.36. The pediatric patients were assigned to the LVH (*n* = 44) and normal geometry (*n* = 138) groups. The LVH group was subdivided based on cardiac geometry [20, 21], including the concentric remodeling (CR; normal LVMI and high RWT, *n* = 24), eccentric hypertrophy (EH; high LVMI and normal RWT, *n* = 16), and concentric hypertrophy (CH; high LVMI and high RWT, *n* = 4) groups.

### Statistical analysis

SPSS 23.0 was utilized for data analysis. Two-sided *P* < 0.05 indicated statistical significance. Continuous data were assessed for normality by the Kolmogorov-Smirnov test. Those with skewed distribution (median and interquartile range *M*(*Q*_1_,*Q*_*3*_)) were compared by the Mann-Whitney U-test. Categorical variates (percentage (%)) were compared by the chi-square (*χ*^2^) test. The Spearman’s correlation coefficient was used to assess the association between two variates. Multivariable logistic regression analysis was performed using stepwise logistic regression with forward selection and backward elimination by removing variables that had a *p*-value greater than 0.05. Odds ratios (ORs) with 95% confidence intervals (CIs) were determined. Receiver operating characteristic (ROC) curves were generated, and areas under the ROC curves (AUCs) were determined to assess the values of indexes in predicting LVH.

## Results

### Demographics, blood pressure, and serum TGF-β_1_ levels of all subjects

Totally, 182 pediatric cases of primary hypertension were included, with 140 boys (76.9%) and 42 girls (23.1%) averaging 12.7 ± 2.4 years old. Of all cases, 134 (73.6%) showed BMI values surpassing the 95th percentile of age- and gender-matched individuals and had a diagnosis of obesity. Of all 182 patients, 57 (31.3%) and 125 (68.7%) showed stages 1 and 2 hypertension, respectively. There were no significant differences between both subgroups based on age, gender, and obesity status. TGF-β_1_ levels were markedly elevated in stage 2 hypertension than in stage 1 hypertension (Table 1). We analyzed the correlations between serum TGF-β_1_ levels and ABPM parameters. Serum TGF-β_1_ amounts had correlations with 24-h SBP and DBP, as well as daytime and nighttime SBP (*P* < 0.05) (Table 2).

**Table 1:** Demographics and serum TGF-β_1_ levels of all subjects

Variables	N	Serum TGF-β_1_ (ng/L)	*Z*	*P*
Age			− 1.244	0.213
School age	41	46.1 (38.6, 48.5)		
Adolescents	141	46.1 (38.8, 51.4)		
Gender			− 1.148	0.251
Male	140	46.4 (38.8, 51.1)		
Female	42	46.1 (38.8, 50.8)		
Hypertension			− 2.376	0.018
Stage 1	57	46.0 (38.6, 48.2)		
Stage 2	125	47.3 (38.8, 52.5)		
Obese			− 1.773	0.076
Yes	134	46.1 (38.8, 52.9)		
No	48	46.1 (38.6, 48.2)		

Bold values represent *P* values less than 0.05

*TGF-β*_*1*_ transforming growth factor β_1_

**Table 2:** Serum TGF-β_1_ levels and ABPM parameters

Blood pressure	*r*	*P*
24-h SBP	0.354	0.0000
Daytime SBP	0.296	0.0000
Nighttime SBP	0.352	0.0000
24-h DBP	0.163	0.028
Daytime DBP	0.074	0.331
Nighttime DBP	0.146	0.054

Bold values represent *P* values less than 0.05

*TGF-β*_*1*_ transforming growth factor β_1_, *SBP* systolic blood pressure, *DBP* diastolic blood pressure

### Serum TGF-β_1_ levels and LVH

We compared baseline characteristics between the LVH and normal cardiac geometry groups. Of all 182 patients, 44 (24.2%) had LVH, and 138 (75.8%) had normal cardiac geometry. These two groups had similar ages, gender distributions, and blood glucose, blood lipid, plasma renin activity, angiotensin II, and aldosterone levels. The results showed that BMI, 24-h SBP, daytime and nighttime SBP, and nighttime DBP were markedly different between the two groups (*P* < 0.05), and TGF-β_1_ amounts were significantly elevated in the LVH group compared with the normal geometry group (51.7 (46.1, 54.9) vs. 46.1 (38.7, 48.1) ng/L, *Z* =  − 4.324; *P* = 0.0000) (Table 3). Serum TGF-β_1_ had a positive correlation with LVH (*r* = 0.321, *P* = 0.0000). TGF-β_1_ amounts were also similar in the CR, EH, and CH groups (48.1 (38.6, 54.8) vs. 52.5 (49.0, 56.0) vs. 54.0 (42.6, 56.4) ng/L; *P* = 0.297).

**Table 3:** Comparison of demographic and laboratory characteristics, ABPM parameters, and serum TGF-β_1_ levels in LVH group and normal geometry group

Variables	LVH group (*n* = 44)	Normal geometry group (*n* = 138)	*P*
Age (year)	12.7 ± 2.5	12.7 ± 2.3	0.947
Gender(male)	34	106	0.564
BMI (kg/m^2^)	30.6 ± 4.9	26.3 ± 4.0	0.0000
24-h SBP (mmHg)	136 ± 11	131 ± 10	0.003
24-h DBP (mmHg)	74 ± 8	72 ± 7	0.142
Daytime SBP (mmHg)	139 ± 12	134 ± 11	0.018
Daytime DBP (mmHg)	75 ± 8	74 ± 7	0.225
Nighttime SBP (mmHg)	131 ± 12	125 ± 10	0.001
Nighttime DBP (mmHg)	71 ± 8	67 ± 7	0.016
Glucose(mmol/L)	4.5 ± 0.5	4.6 ± 0.7	0.444
TG (mmol/L)	1.3 ± 0.6	1.3 ± 0.7	0.840
CHOL (mmol/L)	4.0 ± 0.7	4.0 ± 0.8	0.653
Renin activity (ug/(L.h))	2.7 (1.6, 4.9)	2.7 (1.3, 5.3)	0.633
Angiotensin II (ng/L)	83.9 (69.2, 97.6)	87.9 (76.3, 103.3)	0.243
Aldosterone (ng/L)	98.6 (67.5, 179.4)	93.2 (54.2, 151.0)	0.205
TGF-β_1_ levels (ng/L)	51.7 (46.1, 54.9)	46.1 (38.7, 48.1)	0.0000

Bold values represent *P* values less than 0.05

*SBP* systolic blood pressure, *DBP* diastolic blood pressure, *TG* triglyceride, *CHOL* cholesterol, *TGF-β*_*1*_ transforming growth factor β_1_

### LVH prediction in pediatric patients with primary hypertension

**Fig. 1:**
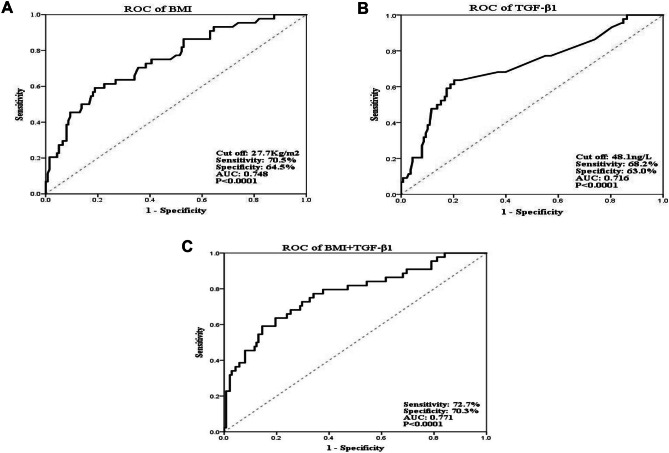
ROC curve analysis of independent risk factors for LVH with primary hypertension: **A** BMI, **B** TGF-β_1_, and **C** BMI + TGF-β_1_

BMI and high serum TGF-β_1_ levels independently predicted LVH (OR > 1, *P* < 0.05) as shown in Table 4. The predictive values of the latter indexes in pediatric LVH were determined from ROC curves. The AUCs for LVH prediction were 0.748 and 0.716 for BMI and TGF-β_1_, respectively. The cutoff values for BMI and serum TGF-β_1_ were 27.7 kg/m^2^ and 48.1 ng/L, respectively (Table 5). In a multivariable logistic regression model combining BMI and TGF-β_1_, an AUC of 0.771 was obtained for LVH prediction, with sensitivity and specificity of 72.7% and 70.3%, respectively (Fig. 1).

**Table 4:** Multivariable logistic regression analysis for LVH in children with primary hypertension

Variables	LVH group (*n* = 44)	Normal geometry group (*n* = 138)	*P*	OR	95% CI
BMI, $\overline{x }$ ± s, kg/m^2^	30.6 ± 4.9	26.3 ± 4.0	0.0000	1.188	1.082–1.305
TGF-β_1_, *M*(*Q*_1_,*Q*_3_), ng/L	51.7 (46.1, 54.9)	46.1 (38.7, 48.1)	0.009	1.063	1.016–1.113

Bold values represent *P* values less than 0.05

*BMI* body mass index, *TGF-β*_*1*_ transforming growth factor β_1_, *LVH* left ventricular hypertrophy, *OR* odds ratio, *CI* confidence interval

**Table 5:** The cutoff values of prediction LVH with BMI and TGF-β_1_

	AUC	SE	95% CI	Sensitivity	Specificity	*P*
BMI	0.748	0.043	0.665–0.832	70.5%	64.5%	0.0000
TGF-β_1_	0.716	0.047	0.624–0.808	68.2%	63.0%	0.0000

Bold values represent *P* values less than 0.05

*BMI* body mass index, *TGF-β*_*1*_ transforming growth factor β_1_, *CI* confidence interval

## Discussion

Primary hypertension is a progressive cardiovascular disease, and vascular remodeling in hypertension continually occurs during the dynamic progression of vasoconstriction, inflammation, fibrosis, and hyperplasia [22]. In the early stage of hypertension, the sympathetic nervous system and RAAS in patients are over-activated, and vasoactive substances such as adrenaline and angiotensin II have elevated levels, resulting in constant increase in the heart rate, elevated blood pressure, and accelerated remodeling of cardiovascular system [23]. Meanwhile, the regulation of neurohumoral mechanism and other factors results in circadian rhythm changes in blood pressure, which are more likely to damage the patients’ heart, kidney, and other target organs [24].

TGF-β_1_, a cytokine displaying multiple functions in fibrogenesis and hemodynamics [25], contributes to hypertensive vascular remodeling by affecting hemodynamics in the body, stimulating cytoendothelin production or RAAS activation [3, 4]. Experimental and clinical data indicated TGF-β_1_ is involved in BP elevation [14, 26]. TGF-β_1_ levels are significantly elevated in cases with hypertension, and upregulation of TGF-β_1_ is associated with cardiovascular alterations [27, 28]. In this study, TGF-β_1_ levels were markedly elevated in stage 2 hypertension compared with stage 1 hypertension, suggesting TGF-β_1_ showed a positive correlation with blood pressure progression.

Children with primary hypertension have specific clinical characteristics, which can be associated with early subclinical TOD [29–31]. Myocardial remodeling in hypertensive cases is defined as heart hypertrophy and fibrosis. LVMI and RWT represent critical indicators for evaluating early LVH [12]. LVH often constitutes the major sign of early heart injury in pediatric hypertensive cases. Representing an intermediary phase of cardiac damage between childhood and adulthood, early cardiac damage is critical for disease evaluation [32, 33]. In this study, of all 182 patients, 24.2% had LVH, indicating that some children with primary hypertension already had early cardiac damage at presentation.

Previous studies have shown that the etiological mechanism of LVH is complex, involving many factors such as intracellular and external stimuli, genes, and heredity.

It was found that TGF-β_1_ is positively correlated with LVMI and hypertrophy in adults [34]. TGF-β_1_ is the cytokine most potently regulating LVH and promoting fibrogenesis. Multiple reports have demonstrated a close association of serum TGF-β_1_ with LVH [35, 36]. TGF-β_1_ is produced in cardiomyocytes and myocardial fibrocytes, as one of the most important cytokines regulating myocardial hypertrophy. First, TGF-β_1_ is the initial factor in the synthesis and deposition of collagen fibers and other extracellular components, which stimulates the synthesis of new contractile proteins in the myocardium, thereby inducing the re-expression of embryonic genes. Second, TGF-β_1_ regulates the synthesis of extracellular matrix proteins and increases the production of collagen, proteoglycan, and fibronectin, which blunts matrix degradation by decreasing collagenase synthesis and upregulating protease inhibitors.

As shown above, elevated TGF-β_1_ was associated with LVH. Serum TGF-β_1_ levels were elevated in LVH cases compared with non-LVH cases. Elevated serum TGF-β_1_ level was positively associated with LVH in children with primary hypertension and represented a contributing factor in LVH. ROC curve analysis showed serum TGF-β_1_ might constitute a valuable molecular marker of LVH in children with primary hypertension. Multivariate logistic regression analysis showed that BMI and TGF-β_1_ may be independent risk factors for LVH. The combination of BMI and TGF-β_1_ had a certain diagnostic and predictive value for LVH in children with primary hypertension, which could provide a new reference index for early clinical identification of hypertensive cardiac damage.

## Limitation

Limitations of the study lie in that, firstly, this was a small-sample single-center study, which requires multicenter trials for validation. Secondly, children with normal blood pressure were not included as the control group. Thirdly, because of no follow-up, the long-term effects of TGF-β_1_ in children with primary hypertension could not be examined. The above cases should be further examined for an extended period of time, particularly monitoring TGF-β_1_ amounts, which may help clearly reveal the exact effects of TGF-β_1_ on BP and LVH.

## Conclusion

In conclusion, the current data revealed an association of serum TGF-β_1_ with BP in children with primary hypertension. Serum TGF-β_1_ content was positively correlated with hypertensive cardiac damage. Serum TGF-β_1_ might represent an important molecular marker for LVH prediction in children with primary hypertension. The combination of BMI and TGF-β_1_ has a certain diagnostic and predictive value in LVH in children with primary hypertension, which may provide a new reference index for early clinical identification of hypertensive cardiac damage.

## Data Availability

The original data shown in the study are included in the article materials, and the corresponding authors may be contacted for additional enquiries.

## Abbreviations

TGF-β_1_: Transforming growth factor β_1_
RAAS: Renin-angiotensin-aldosterone system
TOD: Target organ damage
PWV: Pulse wave velocity
cIMT: Carotid artery intima-media thickness
LVH: Left ventricular hypertrophy
LVM: Left ventricular mass
BP: Blood pressure
SBP: Systolic blood pressure
DBP: Diastolic blood pressure
CNS: Central nervous system
BMI: Body mass index
ELISA: Enzyme-linked immunosorbent assay
ABPM: Ambulatory blood pressure monitoring
LVIDd: Left ventricular internal dimension
IVST: Interventricular septal thickness
LVPWT: Left ventricular posterior wall thickness
LVMI: Left ventricular mass index
RWT: Relative left ventricular wall thickness
CR: Concentric remodeling
EH: Eccentric hypertrophy
CH: Concentric hypertrophy
ROC: Receiver operating characteristic
